# QCL–IR Spectroscopy for In-Line Monitoring of Proteins from Preparative Ion-Exchange Chromatography

**DOI:** 10.1021/acs.analchem.1c05191

**Published:** 2022-03-30

**Authors:** Christopher K. Akhgar, Julian Ebner, Oliver Spadiut, Andreas Schwaighofer, Bernhard Lendl

**Affiliations:** † Institute of Chemical Technologies and Analytics, Technische Universität Wien, Getreidemarkt 9, 1060 Vienna, Austria; ‡ Institute of Chemical, Environmental and Bioscience Engineering, Technische Universität Wien, Getreidemarkt 9, 1060 Vienna, Austria

## Abstract

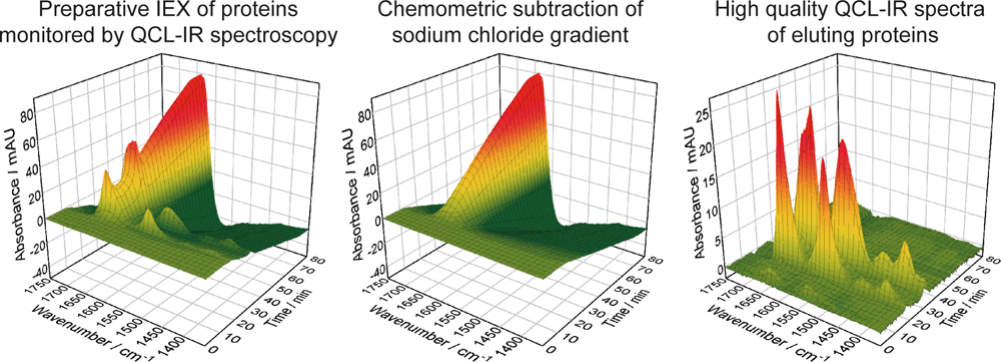

In this study, an external cavity-quantum cascade laser-based mid-infrared (IR) spectrometer was applied for in-line monitoring of proteins from preparative ion-exchange chromatography. The large optical path length of 25 μm allowed for robust spectra acquisition in the broad tuning range between 1350 and 1750 cm^–1^, covering the most important spectral region for protein secondary structure determination. A significant challenge was caused by the overlapping mid-IR bands of proteins and changes in the background absorption of water due to the NaCl gradient. Implementation of advanced background compensation strategies resulted in high-quality protein spectra in three different model case studies. In Case I, a reference blank run was directly subtracted from a sample run with the same NaCl gradient. Case II and III included sample runs with different gradient profiles than the one from the reference run. Here, a novel compensation approach based on a reference spectra matrix was introduced, where the signal from the conductivity detector was employed for correlating suitable reference spectra for correction of the sample run spectra. With this method, a single blank run was sufficient to correct various gradient profiles. The obtained IR spectra of hemoglobin and β-lactoglobulin were compared to off-line reference measurements, showing excellent agreement for all case studies. Moreover, the concentration values obtained from the mid-IR spectrometer agreed well with conventional UV detectors and high-performance liquid chromatography off-line measurements. LC–QCL–IR coupling thus holds high potential for replacing laborious and time-consuming off-line methods for protein monitoring in complex downstream processes.

Preparative liquid chromatography (prep-LC) remains an essential unit operation in downstream processing of complex biopharmaceuticals.^1^ The most widespread detection method for monitoring the corresponding protein concentrations is single-wavelength UV/vis spectroscopy, offering a broad dynamic range and excellent sensitivity. The obtained univariate signal, however, does not give any information about the protein structure and purity during elution.^2^ Thus, the collected fractions have to be additionally analyzed by laborious and time-consuming off-line methods in order to measure critical quality attributes (CQAs). Commonly used off-line methods are high-performance liquid chromatography (HPLC), sodium dodecyl sulphate-polyacrylamide gel electrophoresis, Western blotting and biological activity assays, such as enzyme-linked immunosorbent assays. In recent years, quality by design (QbD) principles were established in the pharmaceutical manufacturing sector.^3^ In order to comply with these regulatory guidelines, process analytical technology (PAT) tools are required, allowing for in-process control and timely adaption of process parameters. Therefore, analytical methods able to provide information about CQAs in an in-line or on-line measurement setup are required.^4,5^

Mid-infrared (IR) spectroscopy provides detailed information about rotational–vibrational transitions of proteins. The established technique in this spectral region is Fourier transform infrared (FTIR) spectroscopy, which is routinely used for quantitative and qualitative analysis of infrared absorption spectra. The most important mid-IR regions for protein quantification and secondary structure determination are the amide I (1700–1600 cm^–1^)^6,7^ and amide II (1600–1500 cm^–1^)^8^ band. Coupling of IR spectroscopy and LC were successfully demonstrated,^9,10^ in most cases using organic solvent gradients. However, flow-through mid-IR transmission measurements of proteins in aqueous matrix have several challenges. One of these constraints arises from the overlap of the HOH-bending band of water near 1643 cm^–1^ with the protein amide I band. A second limitation is the low emission power provided by thermal light sources (Globars) that are used in FTIR spectrometers. Thus, using FTIR spectroscopy, the optical pathlength for aqueous protein solutions is restricted to <10 μm for transmission measurements in order to avoid total IR light absorption of water.^11,12^ These low path-lengths are unsuitable for in-line flow-through measurements due to low robustness and limited sensitivity. Consequently, mid-IR spectroscopy found its way into in-line detection of preparative protein chromatography effluents only recently.^13,14^ In these studies, attenuated total reflection-FTIR spectroscopy was applied, offering robust spectra acquisition, but limited sensitivity. As an alternative approach, complex solvent-removal setups were developed in order to enable protein secondary structure analysis in LC effluents.^15^ Here, the eluent is evaporated, while the analyte is (almost) simultaneously deposited onto a suitable substrate prior to FTIR spectra acquisition. Typical challenges for solvent–evaporation interfaces are, however, the morphology of certain analytes that can change over time and possible spatial inhomogeneity.^9^ Moreover, this destructive type of sample preparation prevents fractionation of the effluent after detection.

Quantum cascade lasers (QCLs) are new light sources in the mid-IR spectral region that provide polarized and coherent light with spectral power densities higher by a factor of 10^4^ or more compared with thermal light sources.^16,17^ In combination with an external cavity (EC), QCLs enable tuning over several hundred wavenumbers, thus offering high potential for mid-IR transmission spectroscopy of liquids. It was shown that the high available spectral power of EC-QCLs opens a wide range of applications, including robust in-line detection of LC effluents.^18^ For protein analysis, the higher spectral power densities enabled to increase the optical path lengths for transmission measurements by a factor of 3–4, thus considerably improving the ruggedness by significantly lowering the backpressure at the cell.^19^ In this framework, academic setups were reported that applied EC-QCLs to investigate the amide I^20,21^ and amide I + amide II band,^22,23^ finally achieving a limit of detection almost 10 times lower than high-end FTIR spectroscopy at similar measurement conditions.^24^ These setups were successfully combined with different chemometric methods in order to quantify individual protein content in complex mixtures^25−28^ and to monitor changes in the protein secondary structure after denaturation.^21,29,30^ Most recently, a commercial EC-QCL-based spectrometer (ChemDetect Analyzer, Daylight Solutions) was introduced, offering robust and sensitive spectra acquisition across the wavenumber region between 1350 and 1770 cm^–1^.^31^

Another challenge in in-line LC-IR is the compensation of possible eluent absorbance bands, which can be higher by several orders of magnitude compared to actual analyte bands. Even though direct subtraction of a background spectrum is possible under isocratic conditions, background correction during gradient elution is more challenging. For this purpose, a method based on a “reference spectra matrix” (RSM) was introduced.^32^ In this approach, the spectra acquired in-line during the LC run are viewed as “sample matrix” (SM), whereas the RSM is recorded during a blank run or the re-equilibration phase of the system. Based on this acquired information, spectral regions that show absorbance bands characteristic for eluent composition are identified, which are located in different spectral regions when compared to the analyte spectrum. Consequently, each spectrum in the SM is corrected with the RSM spectrum that has the closest eluent composition. This method was successfully applied for gradient compensation in a wide range of different reversed-phase HPLC-FTIR applications, including analysis of carbohydrates,^33,34^ nitrophenols,^35,36^ and pesticides.^32,37^

In the present work, the ChemDetect Analyzer was coupled to a lab-scale prep-LC system. The large optical path length of the transmission cell of 25 μm enabled in-line monitoring of proteins without solvent evaporation steps. This LC–QCL–IR coupling was employed to analyze systems based on ion-exchange chromatography (IEX) and different NaCl gradient profiles. For this application, the laser-based spectrometer offers advantages regarding acquisition of protein spectra in aqueous solution. However, due to the limited spectral region of QCL-IR spectra, gradient compensation is more challenging than with FTIR spectra, which cover the entire mid-IR region. In the studied analytical problems, absorption bands from the salt gradient highly overlap with protein absorptions. Three different case studies were performed, covering various real-life conditions used in chromatographic protein downstream processing. In Case I, a reference blank run was directly subtracted from a sample run with the same linear gradient. For Case II and Case III, two different gradient profiles were employed when compared to the one of the reference blank run. Here, a modified RSM-based approach was devised by incorporating the signal of the conductivity detector. With this approach, each spectrum in the SM was corrected with the RSM spectrum that had the closest conductivity value. Thus, a singular measurement of the RSM blank run was sufficient to correct sample runs with different salt gradient profiles, revealing high quality protein spectra. The obtained results demonstrate the successful coupling of a laser-based IR spectrometer with a LC system and present a novel approach for LC-IR-based gradient correction of highly overlapping eluent and analyte mid-IR spectra, indicating high flexibility for future in-line monitoring of the protein secondary structure in chromatographic effluents.

## Experimental Section

### Reagents and Samples

Tris, hydrochloric acid (HCl), and NaCl used for eluents of the prep-LC were purchased from Carl Roth (Karlsruhe, Germany). Hemoglobin (Hemo) from bovine blood and β-lactoglobulin (β-LG) from bovine milk (≥90%) were purchased from Sigma-Aldrich (Steinheim, Germany). For LC-IR and protein secondary structure reference measurements, proper amounts of lyophilized protein powder were dissolved in the corresponding buffer. Ultrapure water (MQ) was from a Milli-Q system from Merck Millipore (Darmstadt, Germany). HPLC-Grade acetonitrile (ACN) and trifluoroacetic acid (TFA) were both purchased from AppliChem (Darmstadt, Germany).

### LC–QCL–IR Setup

**Figure 1:**
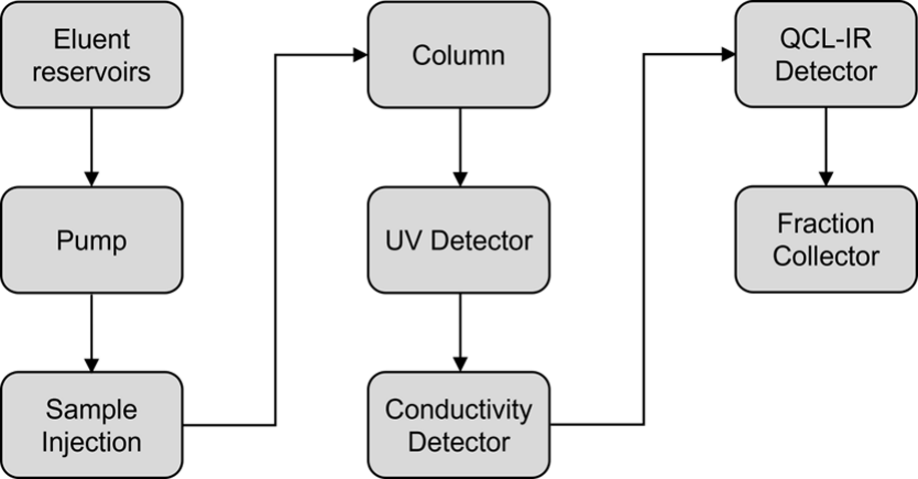
Schematic of the flow path in the LC-QCL-IR setup.

The flow path of LC-IR measurements is illustrated in Figure 1. An ÄKTA pure system (Cytiva Life Sciences, MA, USA) equipped with an U9-M UV monitor, a C9 conductivity monitor, and a F9-C fraction collector was used for the prep-LC runs. All runs were performed with a 1 mL HiTrap Capto Q column (Cytiva Life Sciences, MA, USA). Laser-based mid-IR spectra were acquired with a ChemDetect Analyzer (Daylight Solutions Inc., San Diego, USA). The delay volume between the conductivity detector and ChemDetect Analyzer was determined by injection of 1 M NaCl solution.

### Preparative Chromatography Conditions

For all four prep-LC runs, the setup described in Figure 1 was used. The flowrate was kept constant at 75 cm/h, with Buffer A (50 mM Tris/HCl, pH 8.5) being used for equilibration and wash and a gradient of Buffer A and Buffer B (50 mM Tris/HCl, pH 8.5, 1 M NaCl) being used for elution. Injections were performed using a 1 mL loop, and absorbance at 280 nm as well as conductivity were recorded for all runs. Fractions of 1 mL were collected over the whole run-time and the respective protein concentrations of each fraction measured using the described reversed phase (RP)-HPLC method. The specific elution profiles are shown in Figure S1 and described in detail below.

Blank run: a volume of 1 mL Buffer A was injected. A linear gradient elution was performed over 60 min (30 CVs) from 0% Buffer B to 100% Buffer B.

Case I: as load, 1 mL of 10 mg/mL Hemo and 10 mg/mL β-LG in Buffer A was injected. A linear gradient elution identical to the blank run was performed (0–100% Buffer B in 60 min).

Case II: as load, 1 mL of 5 mg/mL Hemo and 5 mg/mL β-LG in Buffer A was injected. A linear gradient elution was performed over 30 min (15 CVs) from 0% Buffer B to 100% Buffer B.

Case III: as load, 1 mL of 5 mg/mL Hemo and 5 mg/mL β-LG in Buffer A was injected. A step gradient elution was performed with 25% Buffer B 6 min after injection, 50% Buffer B 20.5 min after injection, and 100% Buffer B 30.5 min after injection.

### EC-QCL-Based Mid-IR Measurements

The ChemDetect Analyzer, equipped with an EC-QCL (tunable between 1350 and 1750 cm^–1^) and a diamond transmission flow cell (25 μm optical path length), was used for acquisition of laser-based mid-IR spectra. An external water-cooling unit was operated at 17 °C in order to ensure thermal stabilization of the laser head during operation. Spectra acquisition was performed with the ChemDetect Software package. For flow-through LC-QCL-IR measurements, first, a reference background spectrum was recorded by averaging 121 scans (60 s), followed by continuous acquisition of 20 averaged scans, resulting in a measurement time of 10 s per spectrum. Protein secondary structure off-line reference measurements were acquired by averaging 91 scans during a measurement time of 45 s.

### HPLC Reference Measurements

As an off-line analytical method for the collected fractions, a previously published RP-HPLC method was applied.^38^ In short, a BioResolve RP mAb Polyphenyl column (Waters, MA, USA) was used with MQ as mobile phase A and ACN as mobile phase B, both supplemented with 0.1% v/v TFA. Column temperature was kept constant at 70 °C and an injection volume of 2 μL was used for all samples. Total run time for one injection was 18 min with a flow of 0.4 mL/min and UV/vis absorbance at 214, 280, and 404 nm was recorded for the whole run. A linear gradient from 25% B to 75% B (10 min) was performed, followed by 2 min with 75% B, after which the column was re-equilibrated with 25% B for 6 min.

### Quantification of IR and UV Signals

Protein concentrations (c) across the chromatographic run were calculated from mid-IR and UV signals, according to the Beer–Lambert law
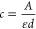
1where *A* denotes the recorded absorbance values, *d* is the transmission path, and ε indicates the molar decadic absorption coefficient.

For laser-based IR spectroscopy, values for A were obtained by integrating the spectra across the amide II region (1500–1600 cm^–1^). The absorption coefficients (ε) for the two proteins were received by integrating the same area region from off-line recorded reference spectra with known protein concentrations.

For quantification of UV/vis spectra, absorption coefficients of the proteins were obtained by performing reference measurements using a Cary 50 Bio UV/vis spectrometer (Agilent Technologies, Santa Clara, California, USA). Spectra were recorded using the Cary WinUV software. Cuvettes with a path length of 10 mm were used to measure 0.5 mg/mL protein solutions. The thereby obtained absorption coefficients at 280 nm agreed well with those calculated via ExPASy ProtParam tool^39^ and were used to calculate protein concentrations.

### Data Processing

Data processing and analysis was conducted with in-house codes developed in Matlab R2020b (MathWorks, Inc., Natick, MA, 2020). During the first preprocessing step, absorbance bands of water vapor from the atmosphere were automatically corrected by subtraction of a water vapour reference spectrum, if necessary. Gradient correction was performed by direct blank run subtraction (Case I) and a modified procedure based on RSM (Case II and Case III).^32^ Finally, absorption spectra were smoothed with a second order Savitzky–Golay filter (window = 15 points). The spectral resolutions of 1.2 and 3.6 cm^–1^ were determined for smoothed and unsmoothed spectra by comparing the bandwidth of water vapor spectra to FTIR reference spectra.

## Results and Discussion

### Relation between Salt Gradient, Conductivity, and Mid-IR Spectra

In-line monitoring of prep-LC effluents was performed with the ChemDetect Analyzer as well as with conventional UV/vis and conductivity detectors (Figure 1). In addition to the routinely recorded signals, laser-based mid-IR spectroscopy offered robust spectra acquisition in the most important wavenumber range for protein secondary structure analysis.

In the present LC–QCL–IR application, IEX was employed as the separation mechanism. Here, the pI of the analyte and the pH of the mobile phase are decisive for the degree of retention. Bound analytes are commonly eluted by utilizing a gradient of increasing salt concentration. Conductivity detection is a widespread method for measuring the salt concentration in IEX.^40^

**Figure 2:**
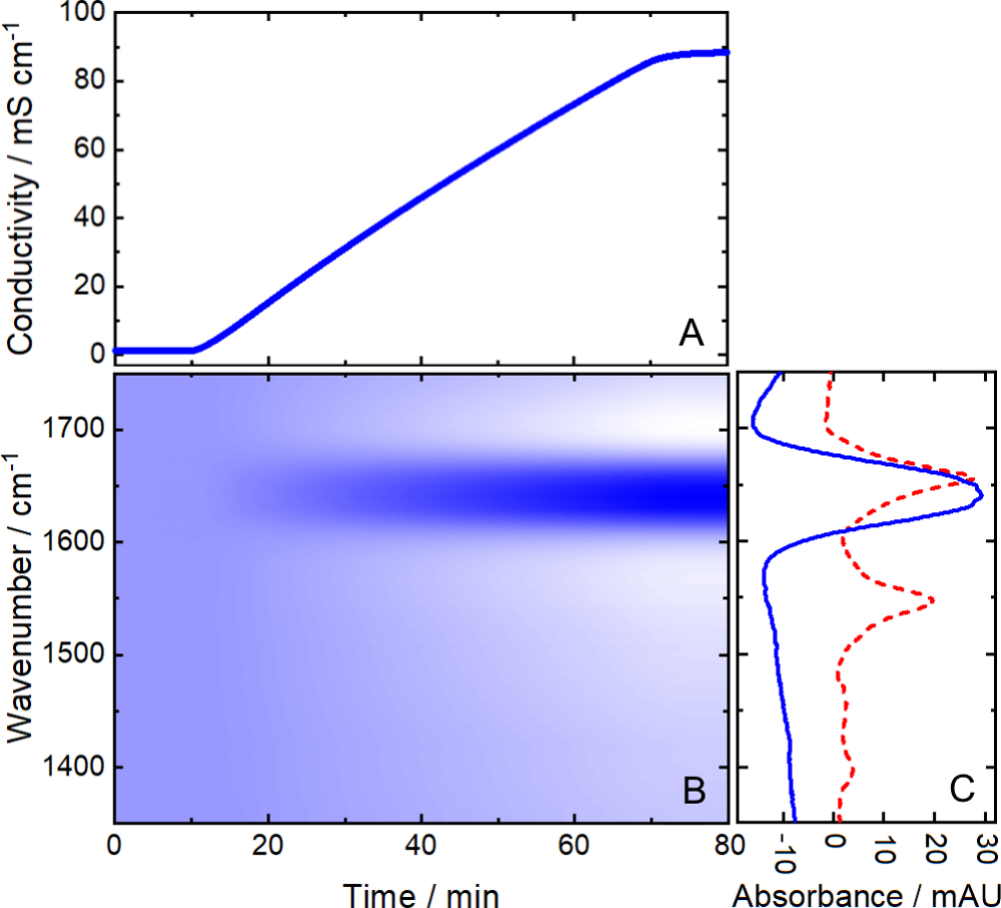
Detector signals from an ion-exchange preparative-liquid chromatography blank run with a NaCl gradient (0–1 M between 10 and 70 min). (A) Conductivity detector response. (B) Heatmap of the corresponding laser-based mid-IR spectra. White areas indicate negative absorbance and dark blue areas positive absorbance. (C) Absorbance spectra of 0.3 M NaCl (retention time: 31.3 min, blue line) and 5 mg/mL hemoglobin (red, dashed line).

Figure 2 displays the signals of the mid-IR and conductivity detector on the example of an IEX blank run with a linear NaCl gradient, and thus, the corresponding signal (A) linearly increases with the NaCl concentration.

The blue line in Figure 2C shows the distinctive mid-IR spectrum of NaCl (blue line) with the NaCl-free starting buffer as the reference. Increasing absorbance can be observed between approximately 1610 and 1680 cm^–1^, while the absorbance in the remaining spectrum decreases with higher salt concentrations. Even though Na^+^ and Cl^–^ are not IR active, reorganization of the water molecules due to the presence of these ions is responsible for a change in the mid-IR spectrum compared to the NaCl-free initial buffer.^41^ The maximum of the HOH bending band of water (at approximately 1643 cm^–1^) is not shifted in position; however, its intensity increases, accompanied by narrowing of the band with increasing NaCl concentrations.^42^ The negative absorbance in the other spectral regions can be related to partial displacement of H_2_O molecules with salt.

Figure 2B shows a 2D heat map depicting the response of the NaCl gradient recorded by the ChemDetect analyzer. White areas indicate negative absorbance, whereas dark blue areas highlight positive absorbance.

The red dashed line in Figure 2C shows the IR spectrum of Hemo, illustrating the spectral overlap of protein bands with the one of NaCl (blue line). Particularly, the amide I band, representing the most important mid-IR region for protein secondary structure determination, is impeded by the modified HOH bending band of water. The described overlap of analyte and changing solvent band indicate significant challenges when employing the ChemDetect Analyzer for in-line monitoring of IEX. In order to obtain feasible mid-IR protein spectra, the influence of the changing salt concentration has to be eliminated. Consequently, feasible gradient compensation strategies are discussed and demonstrated based on model protein systems in the following subchapters.

### Case I: Direct Blank Run Subtraction to Enable In-Line Monitoring of Proteins

In this approach, for background compensation, an analyte-free reference blank run was performed with the same gradient as the sample run. Based on the retention time, the reference spectra were subsequently subtracted from the sample spectra.

**Figure 3:**
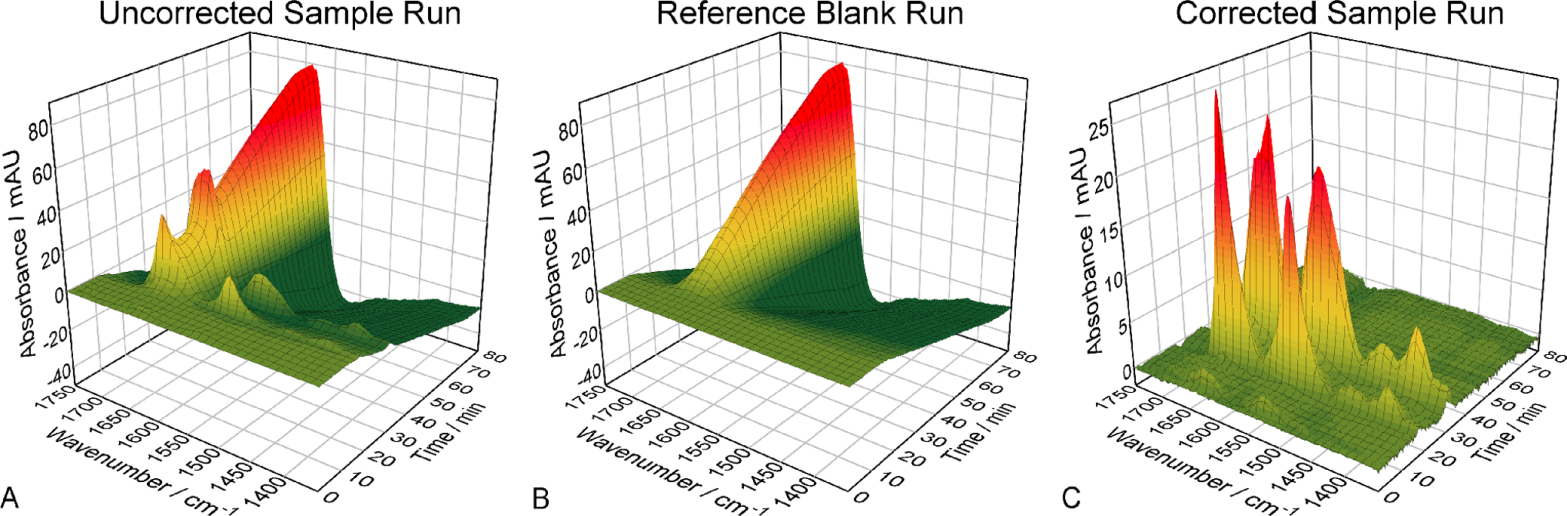
Spectral 3D plots for Case I: (A) uncorrected sample run, (B) reference blank run, and (C) corrected sample run after direct background subtraction.

**Figure 4:**
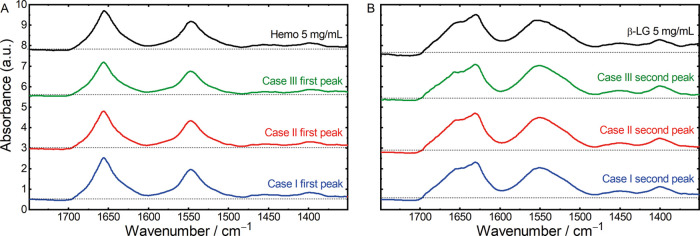
Laser-based mid-IR absorbance spectra, extracted from the (A) first and (B) second peak maxima of the corrected chromatographic runs from Case I (blue lines), Case II (red lines), Case III (green lines), and off-line recorded reference IR spectra (black lines) of 5 mg/mL hemoglobin and β-lactoglobulin. The individual spectra were offset for better visibility. The pointed black lines indicate zero absorbance for every spectrum.

In the sample run, Hemo and β-LG were included, due to their difference in the pI and secondary structure. Figure 3 shows the spectral 3D plots of the (A) uncorrected sample run and the (B) reference blank run, as well as the (C) corrected sample run after direct background subtraction. The mid-IR spectra of the blank run were already discussed above. Even though the amide I and amide II bands are visible in the uncorrected sample run, the effect of the NaCl gradient on the absorbance spectra is clearly dominating. Thus, no reliable information about the protein secondary structure can be obtained. In contrast, the corrected sample run shows a stable baseline and distinct protein spectra. Figure 4 depicts the absorbance spectra extracted from the peak maxima of the differently corrected sample runs as well as off-line recorded reference IR spectra of the investigated proteins. For Case I (blue lines), the first chromatographic peak with a maximum at approximately 17 min can be related to Hemo due to its higher pI of 7.1.^43^ Hemo mainly contains α-helical secondary structures and shows the characteristic narrow amide I band with a maximum at approximately 1656 cm^–1^ and a narrow amide II band at 1545 cm^–1^.^7,44,45^ β-LG is predominantly composed of β-sheet secondary structures^46^ and has a pI of 5.1^47^ and thus elutes second under these IEX conditions. The corresponding absorbance spectra show broader amide I and II bands with β-sheet typical maxima at 1632 and 1550 cm^–1^ and a shoulder at 1680 cm^–1^.^22,48,49^ The obtained high-quality protein spectra thus indicate excellent long-term stability of the ChemDetect Analyzer.

A drawback of this direct gradient compensation approach is the need for a blank run that is performed identical to the sample run. Consequently, any adaptions in the gradient profile require acquisition of an additional blank run and very high reproducibility of the chromatographic system is required in general. The additional time consumption of the blank run and the re-equilibration phase of the column make this approach methodically rigid and impractical to realize for bench-scale as well as industrial applications. Hence, a more flexible strategy is presented in the next subchapter.

### Case II and III: Flexible Gradient Compensation with Adapted RSM

Even though direct blank run subtraction revealed excellent protein spectra, more flexible and less laborious gradient compensation strategies are favorable. Gradient compensation based on RSM requires only a single blank run, which can be used for correcting various gradient profiles.^32^ This approach was successfully applied for HPLC-FTIR, where the entire mid-IR range and eluent specific absorbance bands were available. These specific bands were used as mobile phase identification parameter (IP) in order to select the blank spectrum to be subtracted. This approach, however, is not feasible for monitoring proteins in IEX due to lack of specific absorbance bands and limited sensitivity of FTIR spectroscopy. Due to significantly higher spectral power densities of the EC-QCL, the ChemDetect Analyzer overcomes the typical limitations of FTIR spectroscopy and offers robust and highly sensitive flow-through measurements in the most important wavenumber range for protein analysis.

**Figure 5:**
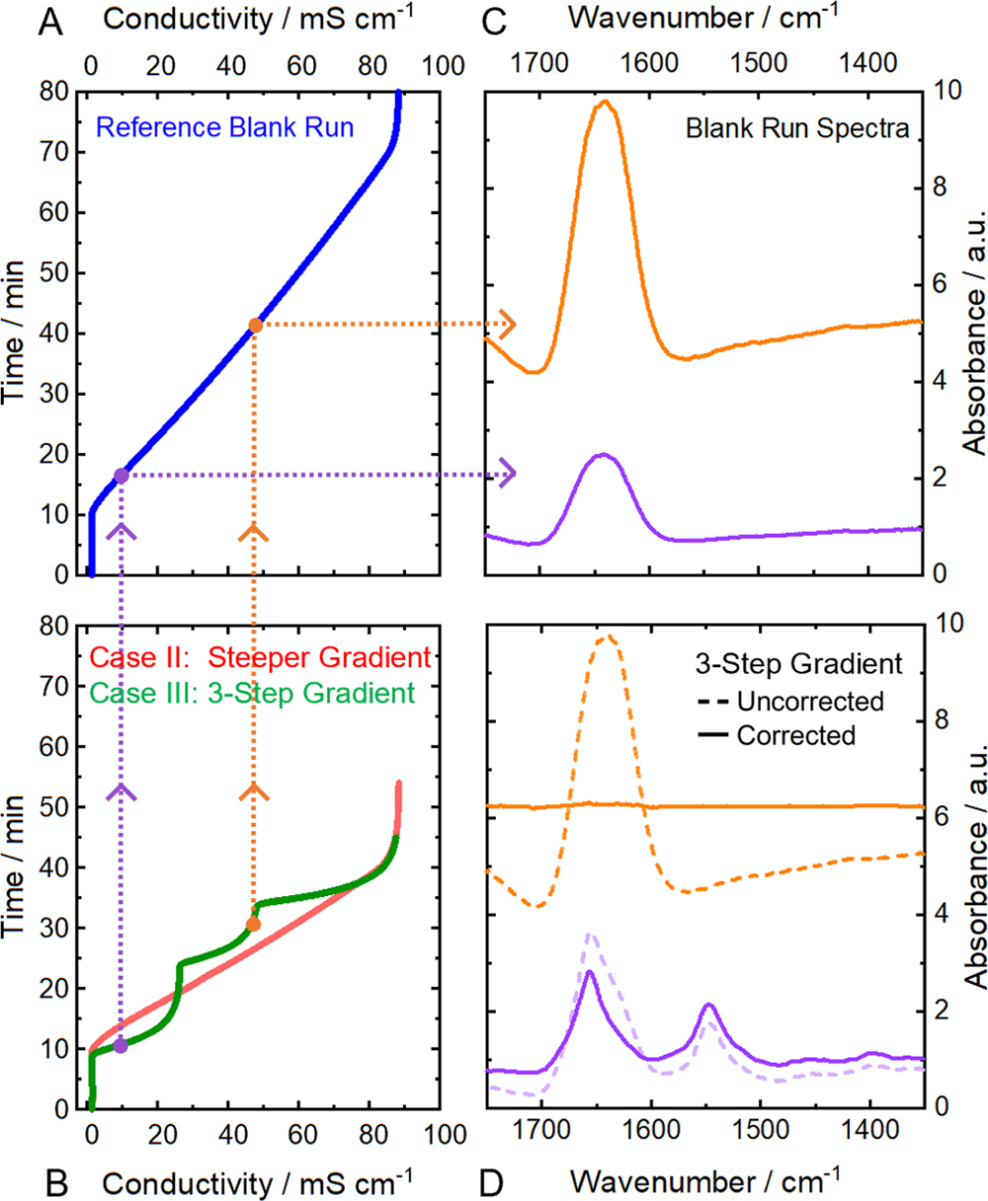
Principle of the applied RSM-based background compensation method. (A) Conductivity detector signals of the reference blank run and (B) sample runs of Case II and Case III. Dotted lines indicate the relation between selected conductivity values and the corresponding mid-IR spectra of background (orange) and protein (purple) of Case III. The absorbance spectra of the (C) blank run were subtracted from (D) the 3-step gradient sample run spectra, based on the most similar conductivity value, revealing distinctive protein spectra (purple) and a flat baseline (orange).

In the present study, a modified RSM method based on an external IP is proposed and successfully applied. For this purpose, the signal from the conductivity detector was related to the mid-IR spectra. The measured conductivity represents an adequate IP, due to its high dependency on the NaCl concentration, whereas the influence of the protein concentration on the conductivity is negligible (Figure S2). For this purpose, the blank run with the linear NaCl gradient (same as in Case I) was taken as the reference. In order to evaluate the applicability of this approach, two chromatographic sample runs with different gradients compared to the one from the reference run were conducted: Case II included a linear gradient with a steeper profile, while Case III featured a 3-step gradient. A specific conductivity value was assigned to each mid-IR spectrum in the SM and RSM, based on the retention time. Figure 5 depicts the principle of the applied compensation method on the example of a baseline and protein spectrum in Case III. Based on the comparison of the measured conductivity in the (C) sample run and (A) the reference blank run, the corresponding (B) blank run spectra are identified. Finally, the (D) sample spectra are corrected by subtracting the identified blank run spectra with the most similar conductivity value.

**Figure 6:**
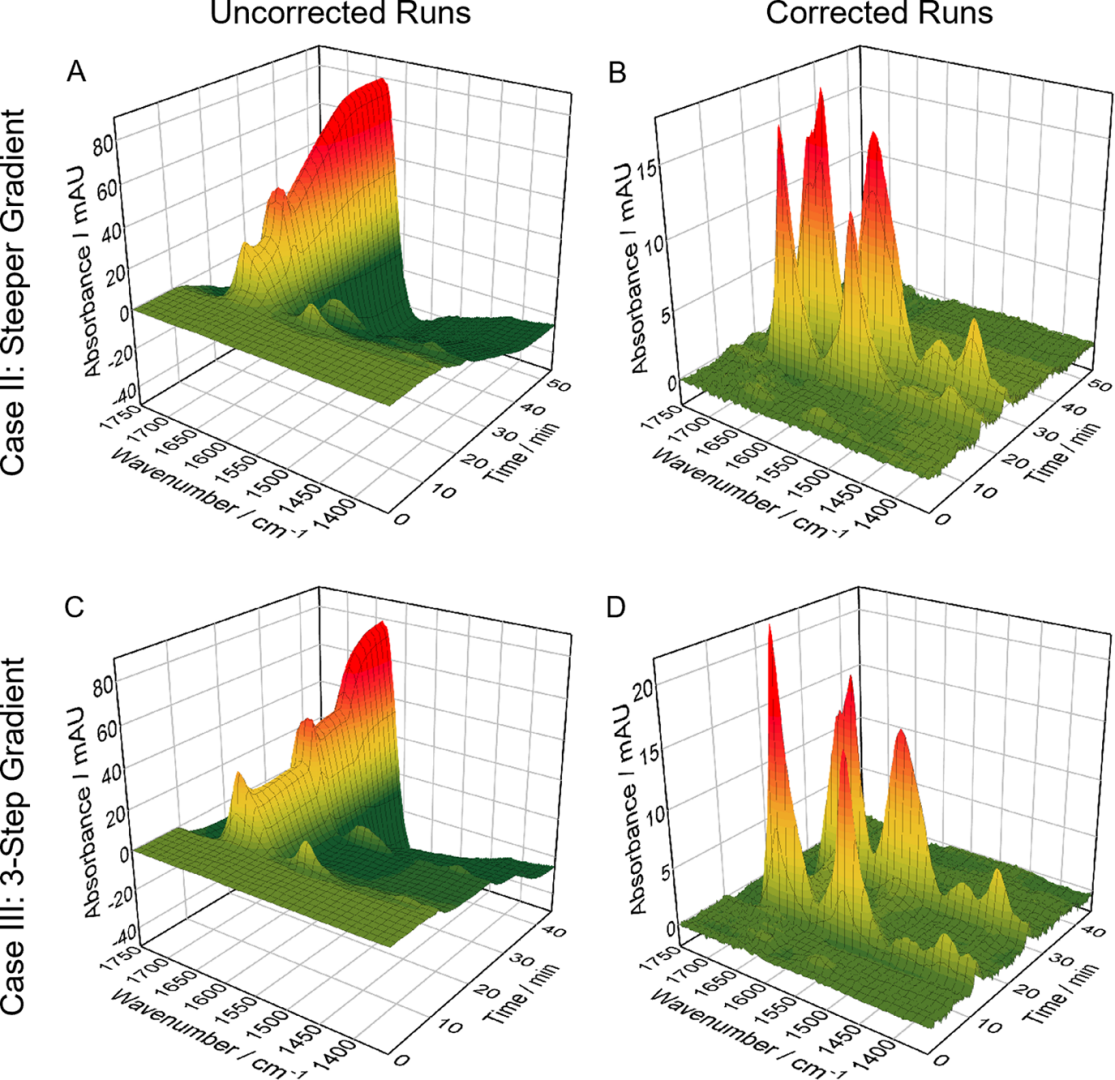
Spectral 3D plots for Case II and Case III: (A,C) uncorrected sample runs and (B,D) corrected sample runs after background correction with adapted RSM.

Figure 6 displays the spectral 3D plots of the uncorrected (A,C) and corrected (B,D) sample runs. The uncorrected run from Case II constitutes a similar profile as the reference blank run with a shorter measurement time, whereas the run from Case III shows a clearly different profile due to the 3-step gradient. Both corrected runs comprise stable baselines and excellent protein spectra, comparable to those from direct blank run subtraction (Figure 4). Consequently, acquisition of a singular blank run is sufficient for correcting sample runs of widely different gradient profiles. Due to high flexibility and little additional time consumption, the applied gradient compensation approach shows high potential for industrial application.

### Comparison of Mid-IR In-Line Measurements to Conventional Off-Line Analysis

**Figure 7:**
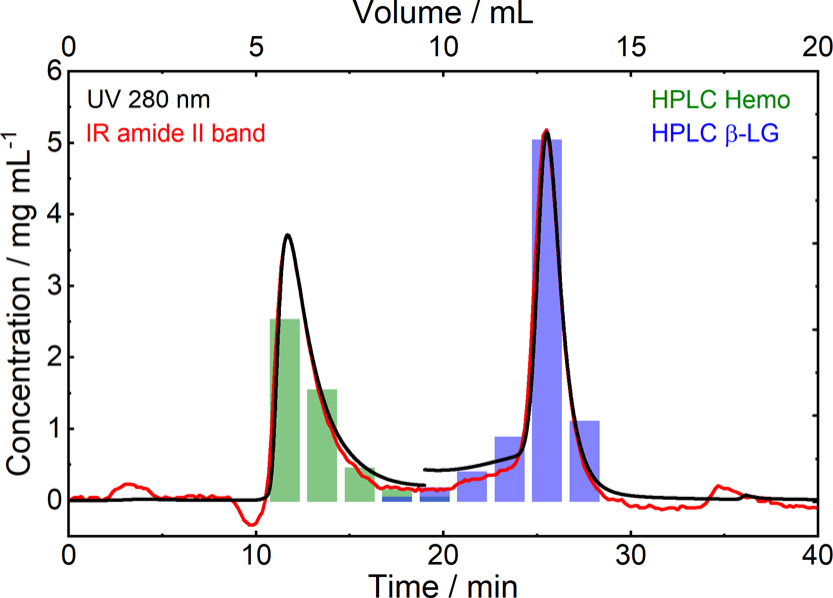
Comparison of protein concentrations obtained from mid-IR amide II band (red line) and UV detector signal at 280 nm (black line) across the chromatographic run from Case III and protein reference concentrations obtained by measuring the collected fractions with reversed-phase HPLC (green and blue bars).

In order to verify the signal of the ChemDetect Analyzer, protein concentrations calculated from mid-IR absorbance were compared to those calculated from the UV detector signal. Figure 7 displays the concentrations, determined from the ChemDetect Analyzer (red line) and UV detector (black line) over the chromatographic run from Case III. In mid-IR spectroscopy, the total protein content is best represented by the amide II band because it is less influenced by water absorption than the amide I band. Thus, the wavenumber region between 1500 and 1600 cm^–1^ was integrated to obtain the absorbance values. For comparison, protein concentrations were also calculated from the UV signals at 280 nm recorded by the UV detector of the LC instrument. UV absorption at this wavelength detects aromatic protein residues and disulfide bonds and is most commonly used to monitor proteins in chromatographic applications.^1^ Absorption coefficients for Hemo and β-LG for IR and UV spectroscopy were obtained from reference measurements with known protein concentrations. Because these values are different for the two proteins, for conversion of the recorded signals to concentration values, the chromatogram was split into two parts and the absorption coefficients of Hemo were used from 0 to 19 min, whereas the coefficients of β-LG were applied between 19 and 40 min. Figure 7 depicts the highly overlapping protein concentrations derived from the signals of ChemDetect and UV detector in the time axis as well as for the protein concentration, demonstrating that equivalent quantitative signals can be obtained by the two methods.

For protein identification, IR spectra from the two chromatographic peak maxima were extracted and compared to pure Hemo and β-LG reference spectra, respectively. Figure 4 reveals excellent agreement between in-line and off-line ChemDetect measurements. Absorbance band positions as well as band shapes show excellent comparability between mid-IR spectra of the chromatographic peak maxima and those of the pure reference solutions. In contrast, with conventional LC instrumentation employing an UV detector, real-time information about the protein secondary structure, and consequently type of protein cannot be obtained. In practical applications where the protein identity or purity are usually unknown, highly biased results would be obtained from direct quantitation of the UV signal because absorption coefficients at 280 nm can be different by more than an order of magnitude between proteins.^50^ Then, protein identification must be performed by off-line RP-HPLC measurements of the collected fractions. In this regard, the green bars shown in Figure 7 indicate the concentration of the protein that was identified as Hemo by RP-HPLC, and the blue bars show the concentration of β-LG. Consequently, the obtained LC-QCL-IR chromatograms agree well with the conventionally applied quantification methods, while offering the additional advantage of providing real-time information about the protein secondary structure.

## Conclusions and Outlook

In this work, a laser-based mid-IR spectrometer was successfully applied for in-line monitoring of proteins from preparative IEX. A major challenge was the highly overlapping absorbance bands of proteins and NaCl gradient that dominated the recorded sample run spectra. Advanced background compensation strategies based on adapted RSM were developed and their implementation in three different case studies resulted in high-quality protein spectra. In Case I, a reference blank run was directly subtracted from a sample run with the same gradient profile. The obtained protein spectra indicated excellent long-term stability of the ChemDetect Analyzer. Case II and III included sample runs with a steeper linear gradient and a 3-step gradient, respectively. Here, a novel gradient compensation approach based on RSM and conductivity IP was introduced. With this method, a single blank run was sufficient for compensating the NaCl gradient in sample runs with distinctively different profiles. The thereby obtained protein spectra showed excellent comparability to off-line reference measurements.

It was shown that the protein concentrations evaluated from signals obtained by the ChemDetect Analyzer are equivalent to UV spectroscopy at 280 nm, which is the standard quantification method for proteins in chromatographic systems. Furthermore, compared to conventional LC detectors, the laser-based mid-IR spectrometer offers the major advantage of providing real-time information about the protein secondary structure, comparable to high-end off-line measurements. The ChemDetect Analyzer thus holds high potential for complementing laborious and time-consuming off-line methods and provides an easily accessible in-line method. In combination with the presented adapted RSM method to compensate for varying gradient profiles, QCL-IR presents a powerful tool for in-process monitoring and control. Especially, in the light of QbD principles, a near real-time PAT tool able to give information about protein secondary structures and corresponding CQAs presents high potential. In the future, LC-QCL-IR coupling can be employed for chemometrics-based analysis of possible impurities and individual quantification of co-eluting proteins.

## Supporting Information Available

Elution profiles of the recorded chromatographic runs, conductivity detector signal of reference blank run and sample run with the same NaCl gradient, and IR spectra and calibration lines for hemoglobin and β-lactoglobulin (PDF)
