# Supplementary material for: QCL–IR Spectroscopy for In-Line Monitoring of Proteins from Preparative Ion-Exchange Chromatography
Source: Anal Chem. 2022 Mar 30;94(14):5583–90. doi: 10.1021/acs.analchem.1c05191 (PMC9008697; doi:10.1021/acs.analchem.1c05191)
Supplement: Supplementary file 1 — ac1c05191_si_001.pdf [file ac1c05191_si_001.pdf]

Supporting information

QCL-IR Spectroscopy for In-line Monitoring of Proteins from Preparative Ion-Exchange Chromatography

Christopher K. Akhgar,[†,‡] Julian Ebner,[§,‡] Oliver Spadiut,[§] Andreas Schwaighofer*[,†] and Bernhard Lendl*[,†]

[†]Institute of Chemical Technologies and Analytics, Technische Universität Wien, Getreidemarkt 9, 1060 Vienna, Austria

[§]Institute of Chemical, Environmental and Bioscience Engineering, Technische Universität Wien, Getreidemarkt 9, 1060 Vienna, Austria

Figure S1. Elution profiles of the performed chromatographic runs.

Figure S2. Conductivity detector signal of reference blank run (red) and sample run (blue)

Figure S3. IR spectra and calibration line for hemoglobin.

Figure S4. IR spectra and calibration line for β-lactoglobulin.

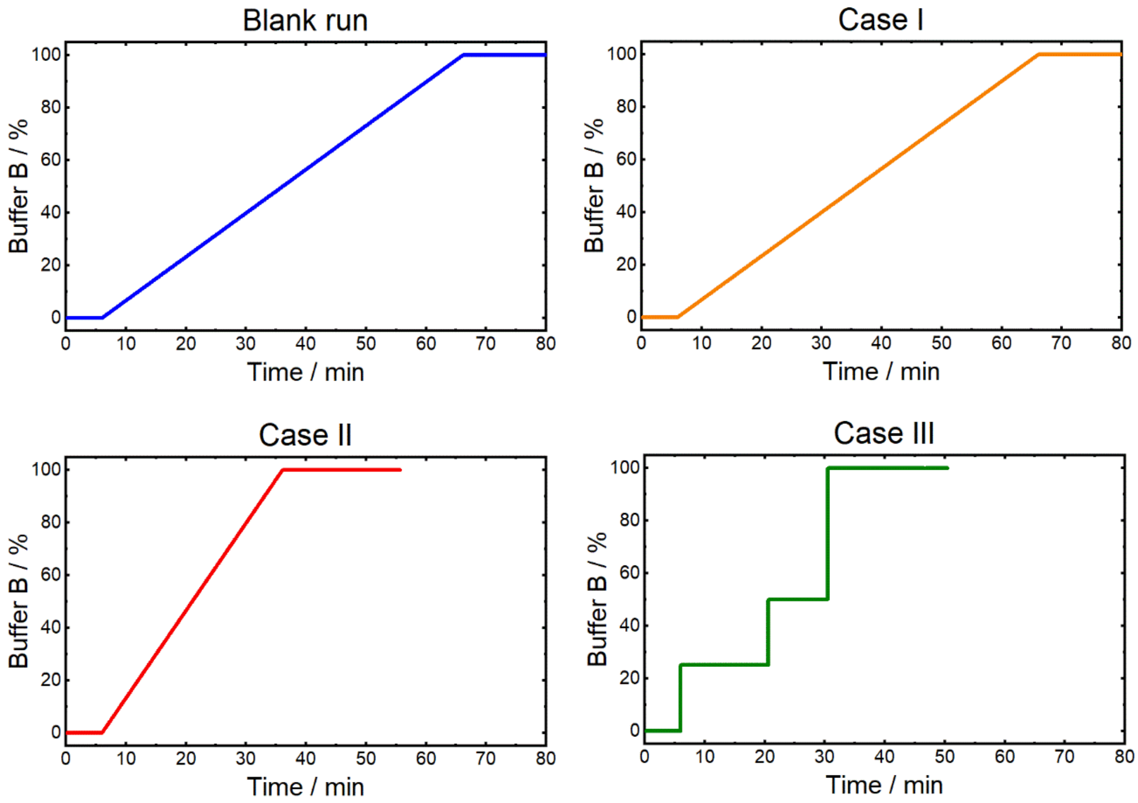

Figure S1. Elution profiles of the performed chromatographic runs.

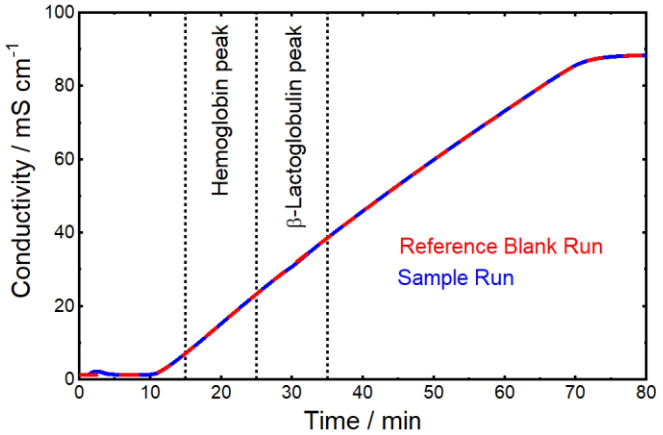

Figure S2. Conductivity detector signal of reference blank run (red) and sample run (blue) with the same NaCl gradient (0-1M between 10 and 70 min). The dotted lines indicate the elution windows of hemoglobin and β-lactoglobulin (only included in the sample run). The conductivity for both runs shows excellent comparability, thus the signal highly depends on the NaCl concentration, whereas the influence of the protein concentration on the conductivity is negligible.

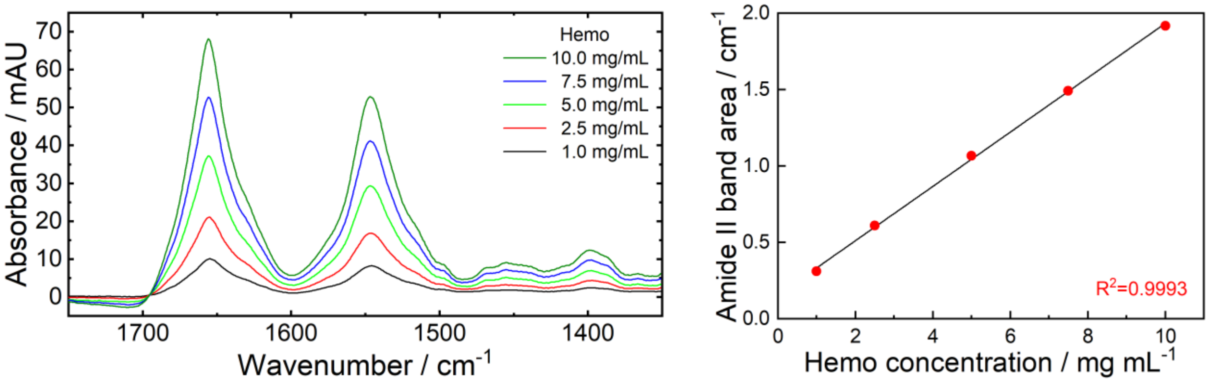

Figure S3. IR spectra and calibration line for hemoglobin.

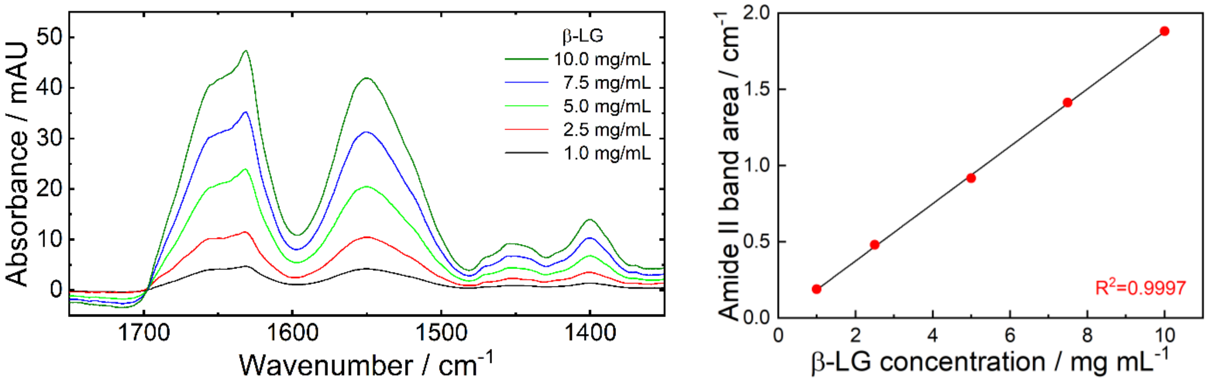

Figure S4. IR spectra and calibration line for β-lactoglobulin.
